# P02.93. Evaluation of blood stasis on tongue diagnosis associated with diabetes mellitus

**DOI:** 10.1186/1472-6882-12-S1-P149

**Published:** 2012-06-12

**Authors:** L Lo, P Hsu

**Affiliations:** 1 Changhua Christian Hospital, Changhua, Taiwan

## Purpose

Diabetes mellitus is a metabolic disorder of hyperglycemia and is associated with the increase in cardiovascular diseases and other complications. In traditional Chinese medicine (TCM), examination of the tongue plays an important role in diagnosis. Tongue color, tongue characteristics (e.g. petechia) and sublingual collateral vessels indicate blood stasis status in TCM. The purpose of this study was to evaluate blood stasis on tongue image and Heart Rate Variability (HRV) associated with type 2 diabetes mellitus (DM).

## Methods

A total of 119 subjects with DM were enrolled for tongue examination and HRV. A high resolution digital camera was used to record tongue images in a standard imaging environment. HRV and pulse wave analysis were performed by ANSWatch. A total of 51 DM patients attended the Quality-based Payment Program for Diabetes Care and received regular health education, examination, and follow-up.

## Results

The average age of the diabetic patients was 61.9±11.2 years. Tongue appearances revealed 23 cases (19.3%) with bluish tongue, 22 cases (18.5%) with petechiae, and 72 cases (60.5%) with engorged sublingual collateral vessels. The bluish tongue revealed significantly higher HRV (p<0.05), higher low frequency (LF) (p<0.05), higher very low frequency (VLF) (p<0.05) and lower total power (p<0.05) compared to the non-bluish tongue. The abnormal sublingual collateral vessels revealed significantly lower high frequency HRV and higher low frequency HRV. There was no significant difference among petechia.

## Conclusion

The bluish tongue, petechia, and engorged sublingual collateral vessels were potential tongue manifestations of blood stasis. Blood stasis in diabetic patients would be important because of potential vascular complications. The relationship between blood stasis and HRV need demonstration by further study.

